# Trigeminal Nerve and Vestibular System: Update on Pathophysiological and Clinical Links

**DOI:** 10.3390/audiolres15060159

**Published:** 2025-11-19

**Authors:** Mario Faralli, Giuseppe Santopietro, Francesco Frati, Luigi Califano

**Affiliations:** 1 ENT Department, University of Perugia, 06156 Perugia, Italy; 2 ENT & Audiology Unit, Department of Neurosciences, University Hospital of Ferrara, 44121 Ferrara, Italy; 3 Department of Audiology and Phoniatrics, San Pio Hospital, 82100 Benevento, Italy

**Keywords:** vestibular system, trigeminal nerve, trigeminal pathologies, vestibular disorders

## Abstract

The points of contact between the vestibular system and the trigeminal nerve remain an active area of research. Anatomically, several connections have been clearly identified, and these may play a role in the development of various disorders. Understanding these connections also proves to be extremely valuable from a clinical perspective. It is increasingly evident that the etiopathogenesis of various vestibular disorders is multifactorial. Therefore, knowledge of the points of interaction between the two systems can assist clinicians in patient assessment and, most importantly, in selecting the most appropriate therapeutic approach. This study is presented as a narrative review. A literature search was conducted to identify studies investigating the correlation between the trigeminal system and the vestibular system, as well as their respective characteristics, to provide a comprehensive overview. Since this is a narrative rather than a systematic review, no specific inclusion or exclusion criteria were applied. So, the aim of this study is to analyze these connections through a comprehensive review of the literature, trying to present a multidisciplinary approach to the topic, one that can involve both the neurologist and the otologist, in order to achieve a more refined management of clinical cases. To better understand their anatomical relationships, we begin by examining the embryological development of both the vestibular system and the trigeminal nerve. Finally, we present current knowledge on the trigeminal influence in certain vestibular disorders—particularly vestibular migraine—and, conversely, the vestibular system’s potential impact on trigeminal-related conditions.

## 1. Introduction

The trigeminal nerves are the fifth pair of cranial nerves. It is the largest cranial nerve and represents the main nerve formed by the first branchial arch [1]. The trigeminal system consists of the trigeminal ganglion and its three branches: ophthalmic, maxillary, and mandibular. The trigeminal nerve has both sensory and motor functions. Peripheral trigeminal receptors are capable of perceiving various sensations and are located in the face, cornea, dura mater, tissues surrounding the mouth and nostrils, mucosal surfaces of the oral and nasal cavities including the paranasal sinuses, teeth, gums, the anterior two-thirds of the tongue, and parts of the external ear [2]. The three branches of the trigeminal nerve communicate with the trigeminal nuclei in the brainstem via the trigeminal ganglion.

The vestibular system is responsible for balance perception and spatial orientation of the body. It forms the posterior portion of the inner ear and consists of the utricle, saccule, and three semicircular canals on each side. The utricular and saccular maculae detect linear movements and the static position of the head, while the three semicircular canals detect angular accelerations. Balance is a complex sense that integrates not only vestibular input but also visual and proprioceptive information [3]. Therefore, the vestibular system works in coordination with other structures to ensure accurate detection and processing of external stimuli.

In 2009, Cuccia A. and Caradonna C. reviewed the correlations between the stomatognathic system, specifically the trigeminal nerve, and body posture [4]. Their study highlighted various factors influencing posture, including emotional aspects, head and neck positions, the oculomotor system, and the vestibular apparatus. The trigeminal system was shown to play a crucial role in postural control mechanisms.

This work aims to summarize the anatomical and embryological connections between the vestibular system and the trigeminal nerve, as well as their involvement in shared pathologies. A thorough understanding of these correlations is essential for clinicians managing balance disorders. It is increasingly clear that posture is influenced by multiple factors and systems. Therefore, a multidisciplinary approach is often necessary to comprehensively assess the patient and design an effective, personalized therapeutic and rehabilitative plan that targets the various systems involved.

## 2. Embryogenesis

We’ll discuss now the embryogenesis of the inner ear and the cranial nerves (particularly the trigeminal and the cochleovestibular nerves), in search of points of contact between these systems that may justify their pathophysiological and clinical connection, which is the focus of our analysis.

### 2.1. Cranial Nerves

The embryogenesis of the central and peripheral nervous systems is a complex process that begins in the earliest days of embryonic development. It starts with a thickening of the ectoderm, known as the neural plate. During the third week of embryonic life, the neural plate forms the neural tube and neural canal through the formation of neural folds. Cells at the lateral edges of these folds give rise to an additional structure called the neural crest. Neural crest cells are characterized by their multipotency, migratory capacity, and ability to differentiate [5]. The neural crest gives rise to various neural and non-neural cells, including melanocytes, cranial skeletal elements, glial cells, and sensory neurons of the cranial nerve ganglia—among them, the trigeminal nerve [6].

Epibranchial placodes, a series of ectodermal thickenings, also contribute to the development of cranial nerves [7]. The neural crest plays a key role in the migration of neurons derived from the epibranchial placodes.

A complex Gene Regulatory Network (GRN) underlies the formation and differentiation of the central nervous system and cranial nerves [8].

The involvement of specific genes in the development of the nervous system and other structures—such as the vestibular apparatus—represents an initial point of connection between functionally distinct systems. The main transcription factors involved in regulating genes at the edge of the neural plate include Bone Morphogenetic Proteins (BMPs), Wingless-related integration site (WNT), Fibroblast Growth Factor (FGF), and HOX genes [5]. Partial inhibition of the BMP pathway—mediated by genes such as *SOX1*, *HES5*, and *SOX2*—is crucial for the formation and development of the neural crest [9]. The WNT pathway is involved in cell’s survival and development, while the FGF pathway promotes proliferation and differentiation of neural crest cells. The Sonic Hedgehog (SHH) signaling molecule directly influences the WNT pathway, and WNT inhibition by SHH has been associated with abnormal development of cranial nerves [10].

Both the neural crest and neural plate contribute actively to cranial nerve development. The main role of neural crest cells in this context seems to be the formation of “corridors” that guide and surround developing neurons [11].

Trigeminal nerve neurons originate from neural crest cells located at the forebrain–midbrain junction and from the trigeminal placode, which is situated near the midbrain–hindbrain boundary, between the prospective eye and ear. The *Pax3* gene has been identified as a marker for trigeminal placode cells. A deficiency in *Pax3* is associated with defective development of the trigeminal nerve [5].

The trigeminal ganglion is composed of an ophthalmic lobe and a maxillo-mandibular lobe, containing both large and small cells. These originate from the placode and the neural crest, respectively. Large cells are primarily somatosensory, while small cells are believed to serve a proprioceptive function for the masticatory muscles [2].

In contrast, the vestibulocochlear nerve arises from both neural crest cells and the otic placode, located at the level of the hindbrain. The otic placode itself derives from the pre-placodal region of the ectoderm.

Transcription factors of the SOX family play key roles in the development of Schwann cells, which support neuronal function by forming the myelin sheath around axons and by providing structural support. *SOX10* activates the transcription factor *Oct6*. The SOX10-Oct6 complex recruits BAF chromatin-remodeling complexes and histone deacetylases HDAC1 and HDAC2. Subsequent activation of *Krox20* leads to chromatin remodeling and the terminal differentiation of myelin [6].

Thus, cranial nerve development is an extremely complex process. In this work, the authors provide a brief overview, highlighting only the major genes involved. However, it is clear the evidence of tight embryological relationships between different cranial nerves, particularly the fifth (trigeminal) and eighth (vestibulocochlear) nerves. Supporting this correlation, Cooper et al. demonstrated the presence of a tyrosine kinase receptor (EphA5) in cranial nerve ganglia V and VIII during development [12].

### 2.2. Inner Ear

The major sensory organs, including the eye and ear, originate from placodes [13]. The inner ear develops from a pre-placodal region, an area of ectoderm located at the anterior edge of the neural plate [14]. By the end of the third week of embryonic development, the otic placode begins to form. This structure is an ectodermal thickening near each neural fold of the future rhombencephalon. Several studies have demonstrated that all cranial placodes arise from a common pre-placodal region [15,16]. The otic placode invaginates to form the otic vesicle (otocyst). By the end of the fourth week, the otocyst separates from the neural plate, becoming an independent structure.

During the second month, the membranous labyrinth begins to develop through elongation and remodeling of the otocyst. The first structures to appear are the utricle, saccule, and the endolymphatic duct, which extends toward the meninges. Two groups of neural cells begin a process of differentiation and specialization: vestibular ganglion cells and spiral ganglion cells.

The simultaneous and organized action of multiple transcription factors is essential for transforming ectodermal cells into the neurosensory cells of the ganglia and for converting the flat epithelium of the saccular region into a tube—the cochlear duct [17]. Numerous transcription factors have been shown to be critical for the development of the neural component of the inner ear (vestibular and spiral ganglia), including Shh, Wnt, and Fgf [18,19,20]. Other important genes are Gata3, members of the Pax family, and Neurog1 (activated by Eya1/Six1) [21].

High levels of Wnt signaling promote the differentiation of tissue into the neural component of the inner ear, while low levels lead to epidermal development [22]. Gata3 and Foxg1 are two genes expressed exclusively in the ear; their absence or mutation is strongly associated with defective development of the neural portion of the inner ear [23,24]. Genes in the Foxi, Gata, Tfap, and Dlx (a Notch ligand) families mainly guide neural tissue differentiation, while Fgf genes serve as signal inducers. IGF1 promotes proliferation within the vestibulocochlear ganglia [19].

Around the seventh week, the semicircular canal primordia begin to form from the utricle. Gpr126, an adhesion G protein-coupled receptor, is essential in the early stages of semicircular canal development [25]. Other genes involved in this process include Wnt and Fgf. Wnt, in particular, plays a role in canal remodeling through cellular reabsorption mediated by Netrin1 at the fusion plate of the canals [26].

By the eighth week, the transformation of the otocyst into the membranous labyrinth is complete. At this point, the ampullae and the utricle and saccule maculae form, concentrating sensory cells. In the cochlea, the organ of Corti begins to develop. Vestibular and cochlear sensory cells converge into the vestibular and spiral ganglia, respectively. The nerve fibers from both ganglia merge to form the vestibulocochlear nerve.

Later, between the third and fifth months, the bony labyrinth forms. Whereas the membranous labyrinth originates from the ectoderm of the otocyst, the bony labyrinth arises from the surrounding mesoderm.

The genes and transcription factors involved in the development of the inner ear—particularly its neural components—are largely shared with those involved in cranial nerve formation, including the trigeminal nerve. As previously noted, the otic and trigeminal placodes appear to derive from a common pre-placodal region, suggesting shared embryological origins.

Thus, despite the anatomical differences between these two structures, their embryogenesis reveals significant similarities that should not be underestimated.

## 3. Anatomy

### 3.1. Peripheral and Cranial Nerve Pathways

There is evidence that trigeminal nerve fibers extend into inner ear structures. Trigeminal ganglion neurons innervate parts of the cochleo-vestibular apparatus, particularly the vasculature. Vass et al. provided direct evidence that trigeminal fibers project to the inner ear’s blood vessels: in guinea pigs, tracer from the trigeminal ganglion labeled fibers around the cochlear spiral modiolar artery, within the stria vascularis, and in the dark cell epithelium of the vestibular crista ampullaris. These fibers surround vessels and secretory cells, indicating a role in inner ear blood flow and ion homeostasis [27].

In addition to inner ear’s vessels, trigeminal sensory fibers innervate parts of the middle ear. Trigeminal ophthalmic-division neurons project to the cochlea, while mandibular-division neurons innervate the middle ear (for example the tensor tympani muscle). These provide somatosensory feedback from the middle ear and even modulate the auditory reflexes. Indeed, somatosensory stimulation can trigger middle ear muscle reflexes, through stimulation, for example, of the external ear or periorbital region (trigeminal territories). The ascending limb of this reflex is thought to involve trigeminal afferents from the external auditory meatus feeding into the ventral cochlear nucleus. The ventral cochlear nucleus then projects to the motor nuclei of tensor tympani (trigeminal motor nucleus) and stapedius (facial nucleus) muscles, modulating middle ear reflexes. Such pathways illustrate peripheral trigeminal–vestibulo–auditory interactions that protect the inner ear. They may also underlie phenomena like somatic tinnitus, where somatic inputs modulate auditory perception [28].

While the vestibular nerve primarily innervates its end-organs, there are hints of peripheral overlap with the trigeminal complex. In animal models, a small connection between the vestibular end-organs and trigeminal system has been noted. For instance, in chickens, a direct projection from the lagena (an otolithic organ in birds) to the spinal trigeminal nucleus was reported [29]. In mammals, direct peripheral convergence is less established, but the proximity of the superior and inferior vestibular ganglia to the trigeminal ganglion in the skull base and the fact that these ganglia share 5HT_1F_ receptors raises the possibility of cross-communication at the ganglionic level, though there is no evidence of direct communication between them [30]. Moreover, studies showed the presence of latency-associated transcript (LAT) of herpes simplex virus type 1 (HSV-1) not only in the trigeminal ganglion but also in the vestibular Scarpa’s ganglion, suggesting a possible route of spread along facio-vestibular anastomotic pathways, as an indirect interconnection of them through accessory routes [31].

In addition, trigeminal motor fibers influence vestibular-related structures. The mandibular division of cranial nerve V innervates the tensor tympani and the tensor veli palatini, which can affect Eustachian tube function and indirectly inner ear pressure dynamics.

While peripheral trigeminal–vestibular interactions in humans are not fully mapped, the animal data confirm that cranial nerve V extends into the ear beyond its classical territory, setting the stage for significant central interactions.

### 3.2. Brainstem and Central Connections

Convergence of trigeminal and vestibular pathways becomes more pronounced in the brainstem.

The trigeminal sensory complex, which includes the principal sensory nucleus in the pons (for touch and pressure), the spinal trigeminal nucleus extending through the medulla into upper cervical cord (for pain and temperature) and the mesencephalic trigeminal nucleus (for proprioception), lies adjacent to the vestibular nuclei in the lateral pons/medulla. Numerous anatomical tracing studies have demonstrated reciprocal connections between trigeminal nuclei and vestibular nuclei.

### 3.3. Trigeminal Afferents to Vestibular Nuclei

Trigeminal primary afferent neurons send some projections directly to vestibular nuclei. In rats, tracer studies showed that fibers from the trigeminal ganglion reach the lateral and superior vestibular nuclei [28]. Marfurt and Rajchert found that a small but consistent contingent of trigeminal primary afferents terminate in the ipsilateral superior and lateral vestibular nuclei. These trigemino-vestibular projections predominantly arise from the mandibular division fibers traveling in the dorsal spinal tract of V [32]. The presence of trigeminal terminals in vestibular nuclei has been confirmed also by others [32,33]. Functional implications include providing somatosensory information to vestibular circuits that coordinate head and body posture [32].

Another route is via the mesencephalic trigeminal nucleus (MesV). MesV is a unique collection of primary sensory neurons (carrying proprioceptive signals from jaw, teeth and extraocular muscles) located within the brainstem. In rodents, MesV neurons (especially those in its caudal portion) project heavily to the vestibular nuclear complex. Pinganaud et al. showed MesV neurons send axons to the medial, inferior, and lateral vestibular nuclei, and to a lesser extent the superior vestibular nucleus [34]. They demonstrated that individual MesV neurons bifurcate to innervate both the vestibular nuclei and the cerebellum (vestibulo-cerebellum) via collaterals. This means that trigeminal inputs (especially proprioceptive) can simultaneously inform vestibular nuclei and cerebellar centers about head/jaw motion, suggesting a role in eye-head coordination [35]. Such projections help link jaw position and movement with balance and eye reflexes. This integration is consistent with observations that vestibular stimulation influences trigeminal motoneurons innervating jaw muscles [36]. Overall, trigeminal inputs (especially proprioceptive) reaching vestibular nuclei represent a feed-forward pathway by which somatosensory signals from the head can modulate vestibular reflexes.

### 3.4. Vestibular Projections to Trigeminal Nuclei

Conversely, neurons in the vestibular nuclear complex project to trigeminal sensory regions. Tract-tracing studies in rats have identified a vestibulo–trigeminal pathway. Valla et al. injected tracers into the trigeminal sensory complex (STC) and found labeled neurons in all major vestibular nuclei (medial, lateral, inferior, and to a smaller extent superior) [37]. Notably, a substantial fraction (30–50%) of these vestibulo–trigeminal projection neurons were GABAergic. This indicates that vestibular nuclei can exert inhibitory influences on trigeminal sensory processing, likely serving a modulatory role. Some vestibular neurons even send branching axons with collaterals to both the trigeminal nuclei and the upper spinal cord (cervical C1–C2, as vestibulo–spinal projections), and these too are mostly GABAergic [37]. This reciprocal connectivity demonstrates that the vestibular system can directly modulate trigeminal sensory pathways, complementing the trigeminal inputs to vestibular pathways.

It is worth noting that in some non-mammalian species, direct connections have also been observed. In frogs, primary vestibular afferent fibers make monosynaptic contacts onto trigeminal jaw motor neurons, and vestibular terminals are found in mesencephalic trigeminal nucleus (abundant) and principal and spinal trigeminal nuclei (sparse) [38,39]. These findings in amphibians support the idea that vestibular information can reach trigeminal circuits at multiple levels (sensory and motor), underscoring an evolutionarily conserved multisensory integration. In mammals, direct monosynaptic vestibular inputs to trigeminal motor neurons are not well documented; rather, vestibular influence on trigeminal motor function appears to be via inter-neuronal circuits in the reticular formation, through a polysynaptic pathway. Nonetheless, trans-neuronal tracing with pseudorabies virus in rats confirmed multi-synaptic pathways linking vestibular nuclei to trigeminal motoneurons in the jaw motor nucleus (via relays in medial vestibular nucleus, caudal prepositus hypoglossi, ipsilateral spinal vestibular nucleus and reticular formation) [36,38]. These central pathways likely mediate vestibulo-motor reflexes such as the vestibulo-masseteric reflex, which consists of a response from the masseter muscles after vestibular stimulation, reflecting activation of trigeminal motor pathways by input from the vestibular nuclei.

### 3.5. Common Brainstem Integrative Centers

Beyond direct V ↔ VIII nucleus connections, there are shared targets where trigeminal and vestibular inputs converge. One key site is the cerebellum. Trigeminal afferents project robustly to the cerebellum, especially the anterior and posterior lobes and the interposed and lateral deep nuclei. Marfurt found trigeminal primary fibers entering the cerebellum via the superior cerebellar peduncle, indicating a predominantly mandibular input to cerebellar circuits involved in coordinating movement [32]. The vestibular system also sends extensive inputs to the cerebellum. Herrick and Keifer’s turtle study showed clear convergence: trigeminal and vestibular nerve both terminate in the cerebellar cortex and deep cerebellar nuclei [40]. Thus, cerebellar neurons can integrate facial somatosensory signals with vestibular balance signals. For instance, during an eye-blink conditioning paradigm in turtles, they observed that trigeminal nerve stimulation and vestibular nerve stimulation converge in the cerebellum, which could underlie synaptic plasticity for associative learning [40]. In humans, this convergence in the cerebellum contributes to fine-tuning reflexes such as the vestibulo-ocular reflex, by incorporating proprioceptive feedback from the head and face.

Other integrative brainstem centers include the reticular formation (RF) and monoaminergic nuclei. Both trigeminal and vestibular collaterals innervate the pontomedullary reticular formation [41]. Additionally, there is evidence that vestibular nuclei project to the locus coeruleus (LC) in the pons, and trigeminal afferents (especially from MesV, carrying proprioceptive information from periodontal ligaments and muscle spindles) also influence LC and other ascending reticular activating system (ARAS) nuclei [42]. These pathways suggest that both systems participate in brain activation and arousal modulation. Sensory signals from head movement (vestibular) or facial inputs (trigeminal) can drive the LC-noradrenergic system, which in turn modulates cortical alertness and attention. For example, chewing or head motion can help maintain wakefulness via LC stimulation. Meanwhile, vestibular inputs to RF and LC may contribute to the autonomic and arousal responses to motion.

### 3.6. Thalamus and Cortex

Ultimately, trigeminal and vestibular information streams converge at higher levels as well.

The thalamus has dedicated relay nuclei for each: trigeminal sensory afferents ascend via the trigemino-thalamic tracts to the ventral posteromedial nucleus (VPM), which relays facial tactile, proprioceptive, nociceptive, and thermal information to the primary somatosensory cortex [43,44]. Vestibular afferents project through vestibulo-thalamic pathways mainly to the ventral posteroinferior nucleus (VPI) and adjacent thalamic regions, such as ventral posterolateral nucleus (VPL), ventral lateral nucleus (VL), VPM and intralaminar nuclei, where they participate in multisensory integration and project to cortical vestibular areas including the posterior insula and parietal operculum. Studies on animals suggest that VPM receives vestibular and trigeminal inputs and is connected to primary and secondary somatosensory cortex [41,42,43,44,45,46,47,48,49,50].

There is overlap and proximity between these thalamic regions, and some neurons in the posterior thalamus respond to both somatosensory and vestibular inputs. This thalamic convergence then feeds into cortical multisensory areas. Cortical integration occurs in regions such as the posterior insula, which is part of the vestibular cortex but also process somatosensory inputs. The insular cortex (IC) has been highlighted as a site of vestibular–somatosensory interaction: patients with posterior insular lesions can exhibit combined deficits in vestibular perception, for example, distorted subjective visual vertical (SVV), and somatosensory functions (for example altered pain/temperature perception). One lesion-mapping study found that stroke patients with insular damage not only had vestibular tone imbalance (tilted vertically), but the severity of this vestibular deficit correlated with abnormalities in thermal/pain sensation, implying a linked processing in the insula [51]. The simultaneous impairment of temperature perception and tilt of SVV suggests that multisensory input converge in the insular cortex. IC is a primary sensory brain region integrating different sensations. Thus, at the cortical level, trigeminal pain/thermal signals and vestibular orientation signals likely converge to contribute to bodily self-awareness and spatial orientation.

Other cortical areas, such as the superior parietal lobule and intraparietal sulcus, also integrate audio/vestibular cues with visual and somatosensory information to construct representations of head and body position in space [31,50].

These widespread overlaps underline that the trigeminal and vestibular systems are not isolated. Even at the highest processing levels, the brain combines their inputs to produce coherent perception and action.

**Figure 1:**
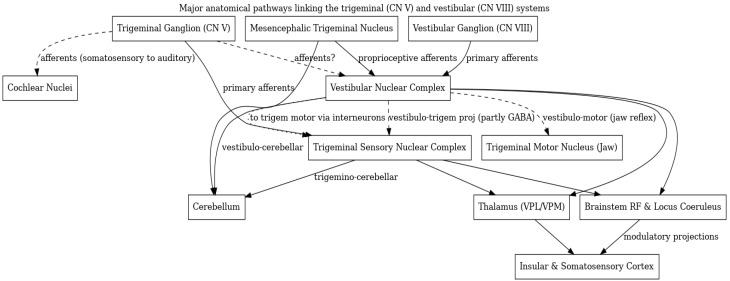
Major anatomical pathways linking the trigeminal (CN V) and vestibular (CN VIII) systems. The trigeminal ganglion (somatosensory neurons) sends primary afferents to the trigeminal brainstem nuclear complex and also has projections to auditory and vestibular brainstem nuclei. A specialized subset of trigeminal primary neurons located in the mesencephalic trigeminal nucleus (MesV, carrying proprioceptive signals) projects to the vestibular nuclei and cerebellum. Vestibular primary afferents from Scarpa’s vestibular ganglion innervate the vestibular nuclear complex; vestibular nuclei in turn send projections to the trigeminal sensory nuclei (including inhibitory GABAergic neurons) and influence trigeminal motor neurons via interneurons (mediating vestibulo-masseter reflexes). Both trigeminal and vestibular nuclei send outputs to common targets such as the cerebellum, thalamus, and integrative brainstem centres (reticular formation and locus coeruleus), which project further to cortical areas (insular and somatosensory cortex). Solid lines denote well-established connections, while dashed lines indicate pathways primarily demonstrated in animal studies or putative connections.

The trigeminal and vestibular systems, classically taught as separate cranial nerve pathways, are in fact deeply interconnected at multiple levels of the nervous system (Figure 1). From the periphery, where trigeminal fibers innervate inner ear structures, to the brainstem, where sensory nuclei exchange projections and jointly innervate reflex circuits, to higher centers in the cerebellum and cortex, there is rich anatomical and functional integration between facial somato-sensation and balance/equilibrium inputs. These interconnections enable the body to coordinate head-eye movements, maintain posture and jaw position, integrate multisensory information for spatial orientation, and modulate sensations like temperature/pain or sound based on head movement context.

The trigeminal and vestibular systems exemplify the principle that no sensory system operates in isolation. The brain’s architecture is one of integration and interaction, leveraging redundant and complementary inputs to enhance function.

## 4. Pathology

### 4.1. Vestibular Migraine and Ménière’s Disease

Among various vestibular disorders, migraine is the condition in which the interaction between the trigeminal nerve and the vestibular system is most evident due his etiopathogenesis. Vestibular migraine (VM) is the leading cause of episodic vertigo in adults and one of the most common diagnoses in children with vestibular disorders [52,53].

In the general population, migraine affects approximately 17% of women and 8% of men, while about 10% of patients with vestibular symptoms have a history of migraine [54]. Vestibular migraine symptoms may be triggered by stress, anxiety, weather changes, and specific foods (e.g., chocolate, cheese). Some patients experience focal neurological symptoms—mainly visual, auditory, and occasionally sensory—known as aura, which precedes or accompanies the migraine attack [55].

The exact etiology of vestibular migraine remains unclear. Several theories have been proposed:

- Vascular theory: proposes that transient cerebral hypoxia from vasoconstriction causes aura, followed by reactive vasodilation that triggers headache [56].
- Trigemino-vascular theory
- Cortical spreading depression theory: attributes the migraine to a wave of neuronal depression across the cortex.
- Neurochemical-vascular theory: suggests abnormal release of vasoactive and pain-inducing substances.

The trigemino-vascular theory posits that trigeminal fibers are activated by algogenic substances, leading to the release of peptides that increase microvascular permeability. Notably, the upper portion of the basilar artery receives sensory innervation from the trigeminal ganglion, while the lower portion is innervated by the dorsal roots of cervical spinal nerves.

The inner ear is vascularized by the anterior inferior cerebellar artery (AICA), a branch of the basilar artery, suggesting that the trigeminal ganglion may also provide sensory innervation to the vasculature of the inner ear. Thus, the trigeminal ganglion may play a significant role in vascular tone regulation, and its dysfunction could contribute to migraine pathogenesis. According to the trigemino-vascular theory, migraine attacks involve the activation of perivascular C fibers [57], which respond to calcitonin gene-related peptide (CGRP) [58]—a potent vasodilator released by trigeminal nerve endings. High plasma levels of CGRP have been detected in the jugular vein during migraine attacks [59].

Several studies support a shared neurogenic control of cerebral and inner ear blood flow, leading to the hypothesis that migraine and vertigo may stem from a common neurovascular mechanism [60]. Additionally, a central connection between the vestibular nuclei and the trigeminal caudal nucleus has been well established [61].

Further research has demonstrated the role of serotonin (5-HT) and its receptors in migraine pathophysiology [62,63]. 5-HT receptors are expressed in trigeminal neurons, the trigeminal caudal nucleus, and cerebral vessels. Co-expression of 5-HT receptors and CGRP has also been observed in all four main vestibular nuclei [64]. CGRP is also involved in the regulation of the otolithic organs, and its loss has been associated with impaired balance [65]. Furthermore, a 2012 study by Xiacheng et al. demonstrated a crucial role of CGRP in motion sickness, identifying an increased CGRP immunoreactivity in the vestibular nucleus of animals affected by motion sickness [66].

Another condition closely linked to trigeminal nerve activity is the Ménière’s disease (MD), which may help explain the association between Ménière’s and vestibular migraine. VM may also evolve into MD, or patients affected by Ménière’s disease may also suffer from migraine.

Numerous studies have explored the association between Ménière’s disease and Vestibular migraine. Some authors report an increased risk of developing migraine in Ménière’s patients and vice versa [67,68].

Among the most widely accepted hypotheses is that dysregulation of the vestibulocochlear vasculature—mediated by the trigeminal nerve and triggered by migraine—leads to inner ear dysfunction via neurovascular connections [69,70]. Other theories propose that vasodilatory neurotransmitters released in migraine may act on inner ear vessels [71,72]. Conversely, the inflammatory state induced by Ménière’s disease might activate trigeminal pathways in the inner ear, triggering migraine symptoms [73]. The relationship between trigeminal nerve activity and these two conditions is thus clearly evident. Meniere’s disease (MD) is characterized by an accumulation of endolymph in the inner ear. According to some studies, the endolymphatic duct may function as a valve to regulate endolymphatic homeostasis [74]. Mechanisms such as inflammation and, more notably, ischemia, which can impair the function of the endolymphatic duct, have been identified as possible causes of Meniere’s disease [75,76]. The shared vascular etiology of MD and vestibular migraine (VM) thus represents an important link between the two conditions. A reversible vasospasm of the internal auditory artery may also explain the vestibular symptoms associated with migraine. This theory could further support a connection between MD and VM [65]. Another possible explanation involves local leakage from the inferior cerebellar arteries or the basilar artery, leading to subsequent activation of the trigeminal nerve [61].

In 2005, Marano et al. showed that nociceptive stimulation of the trigeminal ganglia in migraine patients could induce the appearance or modification of spontaneous nystagmus. This phenomenon may be explained by the presence of proprioceptors in extraocular muscles, which project to the trigeminal system and, in turn, to the brainstem reticular formation, which sends efferent signals back to the extraocular muscles [77].

In the case of Menière’s disease, the connection to VM likely stems from the shared involvement of the trigeminal nerve. Given the established role of the trigeminal system in migraine pathogenesis, it is plausible that anatomical and physiological factors underpin its involvement in Menière’s disease as well.

### 4.2. Other Vestibular and Balance Disorders

The current literature on trigemino-vestibular disorders is limited. Aside from Vestibular migraine and Menière’s disease, no other significant pathogenetic associations between the trigeminal nerve and the vestibular system have been clearly identified to date. However, certain audiological pathologies can present with trigeminal symptoms, particularly expansive lesions.

Tumors located in the cerebellopontine angle are among the conditions that may manifest with trigeminal neuralgia. Large vestibulocochlear schwannomas (typically classified as Koos grade IV) can present with trigeminal symptoms, which are usually due to compression of the trigeminal nerve by the tumor mass [78].

Among cerebellopontine angle tumors, epidermoid cysts are the most frequently associated with trigeminal neuralgia, while vestibulocochlear schwannomas more rarely cause such symptoms. In 2022, Onoda et al. reported a rare case of a small vestibular schwannoma (12 mm in diameter) presenting with asymmetric hearing loss and trigeminal neuralgia. MRI scan imaging revealed that the symptoms were caused by direct compression of the trigeminal nerve, likely due to an anatomical configuration involving reduced distance between the fifth and eighth cranial nerves [79].

Neurovascular compression syndrome (NVCS) is a condition in which cranial nerves are compressed by adjacent blood vessels [80]. Among the most common forms is trigeminal neuralgia, whereas vestibular paroxysmia is considerably rarer. Trigeminal neuralgia is characterized by brief episodes of unilateral pain in the areas innervated by the trigeminal nerve. This pain is typically triggered by low-intensity stimuli [81]. Vestibular paroxysmia, on the other hand, results from compression of the vestibulocochlear nerve by the anterior inferior cerebellar artery. Patients typically experience sudden, brief episodes of vertigo, often occurring multiple times a day—up to 30 episodes per day. Tinnitus may also be present, usually resolving in less than a minute. When present, the tinnitus is distinctive: rhythmic and resembling the sound of a typewriter (“typewriter tinnitus”) [82].

Vestibular paroxysmia and trigeminal neuralgia, along with hemifacial spasm and glossopharyngeal neuralgia, are all classified under the umbrella of NVCS.

## 5. Conclusions

This work aimed to summarize the potential associations between the vestibular and trigeminal systems based on available literature. Clear similarities and direct connections have been identified at both the anatomical and embryological levels. From an embryological standpoint, common transcription factors and genetic elements have been implicated, suggesting a shared lineage. Anatomically, direct connections between vestibular and trigeminal nuclei, as well as interactions with other components of the vestibular system (e.g., the cerebellum), have been thoroughly described in previous studies.

From a pathogenetic perspective, however, the interplay between the trigeminal and vestibular systems remains incompletely understood and warrants further investigation. In the case of vestibular migraine, the involvement of the trigeminal nerve in the pathophysiology of the disease is well established, highlighting a close relationship between trigeminal function and balance control. For other conditions, no clear evidence supports a consistent correlation between the two systems. Therefore, additional studies are needed to better define the potential influence of the trigeminal nerve on vestibular pathologies and to elucidate its role in balance perception.

## Data Availability

Data is contained within the article.
